# One-year predictors of PTSD symptoms, anxiety, and depression in SARS-CoV-2 survivors: psychological flexibility and major life events as main predictive factors

**DOI:** 10.3389/fpsyg.2024.1378213

**Published:** 2024-08-27

**Authors:** Sérgio A. Carvalho, Helena Pinto, Diogo Carreiras, Lara Palmeira, Marco Pereira, Inês A. Trindade

**Affiliations:** ^1^University of Coimbra, Center for Research in Neuropsychology and Cognitive Behavioral Intervention (CINEICC), Coimbra, Portugal; ^2^HEI-Lab: Digital Human-Environment Interaction Lab, School of Psychology and Life Sciences (EPCV), Lusófona University, Lisbon, Portugal; ^3^Instituto Superior Miguel Torga, Coimbra, Portugal; ^4^CINTESIS@RISE, CINTESIS.UPT, Portucalense University, Porto, Portugal; ^5^Center for Health and Medical Psychology (CHAMP), School of Behavioural, Social and Legal Sciences, University of Örebro, Örebro, Sweden

**Keywords:** SARS-CoV-2 survivors, COVID-19 pandemic, PTSD symptoms, anxiety, depression, psychological flexibility

## Abstract

**Introduction:**

The COVID-19 pandemic held considerable health-related outcomes worldwide, including mental health challenges, with elevated risk of psychiatric sequelae.

**Methods:**

This study aimed to test the longitudinal (1 year) predictive role of psychosocial factors on post-traumatic stress disorder (PTSD), anxiety, and depressive symptoms in SARS-CoV-2 survivors (*N* = 209 at T1; *N* = 61; attrition rate 70.83%), through Pearson’s correlation analyses and longitudinal multiple regression analyses. Participants (age *M* = 35.4, *SD* = 10.1) completed online self-report questionnaires of psychosocial variables, PTSD, anxiety, and depression.

**Results:**

Depression and anxiety symptoms were increased, and 42% of survivors presented clinically meaningful PTSD symptoms. PTSD symptoms were longitudinally predicted by having children (*β* = 0.32, *p* < 0.01), number of recent major life events (*β* = 0.34, *p* < 0.01), and psychological flexibility (*β* = −0.36, *p* < 0.01). Number of major life events (*β* = 0.29, *p* < 0.05) and psychological flexibility (*β* = −0.29, *p* < 0.05) predicted anxiety. Number of recent major life events (*β* = 0.32, *p* < 0.01) was the sole predictor of depressive symptoms.

**Discussion:**

Psychosocial variables contribute to the long-term harmful effects of the COVID-19 pandemic on psychopathological symptoms. These results suggest that, during the pandemic, mental health was impacted by both socio-contextual factors and individual self-regulatory skills, namely the ability to respond flexibily to contextual cues and guide behavior according to the direct experience. Specifically, results point out the importance of societal incentives to reduce parental burden and socioeconomic losses, as well as to promote adaptive psychological skills such as psychological flexibility.

## Introduction

The Coronavirus Disease 2019 (COVID-19) global pandemic has resulted in more than 650 million cases confirmed (more than 7 million deaths, and more than 28 thousand in Portugal, reaching its peak of deaths of more than 25 thousand in February 2021) by the end of June 2024. Many countries have implemented measures to contain the spread of the virus (from self-isolation to public health sanitary guidance) that have significantly impacted the lives of populations worldwide (Bedford et al., 2020), with long-term psychopathological symptoms (Hunt et al., 2022), mainly when psychopathology (e.g., depression and anxiety) was already a leading cause of disability and burden globally pre-pandemic (Vos et al., 2020). Indeed, COVID-19 survivors present an elevated risk of developing psychopathological symptoms (Ma et al., 2020), including post-traumatic stress symptoms (PTSD), with significant levels of distress and impairment (Tarsitani et al., 2021). Also, psychopathological symptoms (e.g., depression) were found in patients weeks (Renaud-Charest et al., 2021) and months after recovery (Guideline NG193 N, 2021), mainly, but not exclusively, in cases of severe COVID-19 (Weerahandi et al., 2021). It is suggested that psychopathological vulnerability during the pandemic may not only result from the interplay between inflammatory mediators and neurotransmitters directly resulting from infection (e.g., ‘cytokine storm’; Kempuraj et al., 2020), but also from elevated neuroinflammation indicators of stress (e.g., the psychologically mediated impact of lockdown, social distancing, and overall changes in lifestyle). However, some evidence suggests that symptoms of depression and anxiety decreased during lockdown (e.g., Rehman et al., 2023). This calls for a better understanding of the sociodemographic, interpersonal, and individual psychological factors that contribute to mitigating or exacerbating psychopathological risk during the pandemic.

Studies with multicultural samples found that the perceived threat of COVID-19 is a major contributor to depression, anxiety, and post-traumatic stress (Matos et al., 2021b), with contextual (e.g., financial stability) and psychological (e.g., fear of infection) factors impacting these symptoms (Di Crosta et al., 2020). On the other hand, some studies with samples of young adults suggest that fear of COVID-19 is associated with more adherence to preventive behaviors and thus with more satisfaction with life (Green and Yıldırım, 2022), which calls for the need to further understand the (mal)adaptiveness role of threat-based psychological processes. Parenting children with severe diseases, as well as experiencing stressful life events, seem to be risk factors for psychopathology during the pandemic (Guideline NG155 N, 2020), echoing decades-long psychological research (Kendler et al., 1999). Indeed, COVID-related anxiety is associated with the anticipation of life changes, job loss, and socioeconomic difficulties (Schonfeld et al., 2023), and thoughts about COVID-related death are associated with depression (Fairlamb, 2022). Notwithstanding, resilience (Rossi et al., 2021) and intrapersonal coping mechanisms (Wright et al., 2022) seem to be vital to understanding the vulnerability to and protection against the development of psychopathological symptoms during the pandemic.

Resilience is broadly conceptualized as the ability to bounce back from stressful events and overall adversity (e.g., loss, trauma), resulting in a stable trajectory of psychological health (Bonanno, 2004). During the COVID-19 pandemic, resilience has been brought to the center stage of discussions on mental health (Killgore et al., 2020), and was found to be a crucial factor in protecting against psychopathology and psychological distress and a buffer of the impact of demographic risk factors (e.g., age, gender, education), as well as health-related ones (e.g., chronic illness, SARS-Cov-2 infection, alcohol consumption) (Arslan and Yıldırım, 2021; Tudehope et al., 2022). Nevertheless, although a helpful construct, resilience only provides an overall snapshot rather than a multidimensional picture of the many factors that might contribute to resilience.

Psychological flexibility, defined as the ability to maintain or change behavior according to direct environmental cues and to adapt to situational demands to pursue a valued and meaningful life (Hayes et al., 2006), has been put forward as a critical resilience factor during the COVID-19 pandemic (McCracken et al., 2022), and was found a predictor of better mental health outcomes (Prudenzi et al., 2023) and of chronic anxiety and depression (Hemi et al., 2023) during the COVID-19 pandemic. Based on extensive research on its protective role against psychopathology (Cherry et al., 2021), psychological flexibility is especially relevant during global uncertainty. Indeed, psychological flexibility appears to be cross-sectionally associated with well-being outcomes during the pandemic, is associated with less peritraumatic distress (Kroska et al., 2020), and mitigates the nefarious impact of the COVID-19 lockdown-related risk factors on COVID-19 peritraumatic distress, depression, and anxiety (Pakenham et al., 2020). Although the protective role of psychological flexibility during the COVID-19 pandemic has been extensively studied, samples have been composed of a mix of infected and non-infected participants (see Yao et al., 2023), which may not rigorously grasp the specific impact of psychosocial factors in the mental health of COVID-19 survivors, such as the fear of infecting others, and the shame and guilt associated to ethically tricky decisions (e.g., providing care to loved ones in need) (Haller et al., 2020).

The detrimental impact of shame on mental health in the context of the COVID-19 pandemic has been suggested (Cavalera, 2020), but only a few studies have focused on COVID-related shame. Although clinicians recognize illness-related shame as a relevant health determinant, few studies have empirically explored its negative associations with psychological health in illness populations (Trindade et al., 2018). These associations are potentially more appropriate in an infectious condition such as COVID-19, where individual behaviour is directly accounted for its contraction. Narrative studies suggest that COVID-19 patients may experience internalized shame for having contracted and/or contaminated others (Sahoo et al., 2020), and shame seems to be a mechanism underlying threatening COVID-19 illness perception and psychological distress (Hamama and Levin-Dagan, 2022). These data suggest that the role of COVID-related shame should be duly explored.

The current study aimed to explore the role of contextual (major life events, previous psychiatric diagnoses, job loss) and psychological (COVID-related shame, resilience, psychological flexibility) factors as longitudinal predictors of symptoms of PTSD, anxiety, and depression over 1 year during COVID-19 pandemic, in a sample of Portuguese SARS-Cov-2 survivors.

## Methods

### Participants

The current sample comprises 209 individuals who had been infected with the SARS-CoV-2 virus by the time of the first assessment (T1). Participants were between 19 and 61 years of age and were infected on average 80.02 (*SD* = 68.49) days before data collection, being 17.2% in isolation at the moment of assessment.

Table 1 presents sociodemographic, SARS-CoV-2 infection-related clinical characteristics and psychopathological symptoms. Most SARS-CoV-2 survivors (58%) tested positive during the third wave of the pandemic in Portugal (from the last week of December 2020 to the time of T1 data collection [January–February 2021]), while 4.8% during the first wave of the pandemic in Portugal (March 2020 – September 2020).

**Table 1:** Demographic, clinical and pandemic-related characteristics, and psychopathological symptoms at T1 (*N* = 209).

*Gender, %*	
Men	13.9
Women	86.1
*Age, M ± SD*	35.4 ± 10.1
*Marital status, %*	
Single	45.5
Married or cohabiting	50.2
Divorced	3.8
Widowed	0.5
*Have children, %*	47.8
*Employment status, %*	
Employed	77
Unemployed	11.5
Student	9.6
Working student	1.4
Retired	0.5
*Working from home †, %*	34.9
*Previous psychiatric diagnosis †, %*	30.6
*Comorbid physical illness †, %*	20.6
*Days in isolation, M ± SD*	14.4 ± 25.8
*Lost job due to pandemic †, %*	8.1
*Lost someone to COVID-19 †, %*	10.5
*Perception of symptom severity, M ± SD*	4.89 ± 3.20
*Reported symptoms, %*	
Asymptomatic	11.0
Fever	28.7
Shortness of breath	16.3
Cough	47.8
Decrease or loss of smell	65.6
Decrease or loss of taste	58.4
Muscle aches	57.9
Diarrhea	23.4
*Recovery from COVID-19, %*	
Without prescribed medication	62.2
Required prescribed medication	34.0
Required hospitalization	3.3
Required hospitalization in an intensive care unit	0.5
*Concern about having infected someone, %*	
Never	5.3
Rarely	11.5
Sometimes	17.2
A considerable amount of time	16.7
A lot of time	16.7
All the time	32.5
*Vaccinated †, %*	2.9
*Psychopathological Symptoms*	
PTSD symptoms due to infection, M (SD)	32.90 (13.22)
Normal %	57.9
Possible diagnosis of PTSD %	42.1
Anxiety, *M* (SD)	8.68 (4.31)
Normal, %	42.6
Mild, %	22.0
Moderate, %	30.1
Severe, %	5.3
Depressive symptoms, *M* (SD)	5.80 (4.1)
Normal, %	67.9
Mild, %	13.9
Moderate, %	17.7
Severe, %	0.5

M, mean; SD, standard deviation. † = Dummy variables (Yes = 1; No = 0).

The attrition rate was 70.83% from T1 to T2 (i.e., 12 months after T1). Differences in sociodemographic, clinical, and outcome variables between completers and non-completers are displayed in Supplementary Table S1. Completers and non-completers only significantly differed in marital status (completers had a higher proportion of single and widowed participants) and in the working-from-home variable (completers had a higher proportion of participants working from home).

### Procedures

This study was approved by the Ethics Committee of the Faculty of Psychology and Education Sciences of the University of Coimbra (15/09/2020). The sample was recruited in Portugal through a web-based survey created on LimeSurvey. It was advertised through the study’s social media pages created for this purpose. All participants provided informed consent before proceeding to the inquiry. Participants who wished to be included in the longitudinal study provided their email addresses. The first assessment wave (T1) took place in February 2021, and the second (T2) in February 2022. At T2, participants were contacted by email for follow-up assessment. Inclusion criteria assessed at T1 included being over 18 years old, residing in Portugal, and being able to answer the set of questionnaires in Portuguese. A total of 209 participants completed T1 assessment, and 61 participants completed T2 assessment (29.2%). After T1, 60 randomly picked participants (picked blindly, using their study IDs through https://www.random.org/) were each awarded a voucher for 50€ to be spent in groceries, electronics, or clothing stores. The final revised version of the current paper was proofread with the use of Grammarly Premium, 2024 © Grammarly Inc.

### Pandemic context during data collection

During T1 (February 2021), most Portugal residents were under a second mandatory lockdown that started on January 15, 2021 (the first Portuguese lockdown took place between March and May 2020) and ended on March 15, 2021. The Omicron variant was first identified in Portugal in November 2021. The second assessment (T2) took place in February 2022, when more than half of the population had received the third dose of the vaccine.

### Measures

All participants completed sociodemographic, clinical (e.g., previous SARS-CoV-2 infection, previous psychiatric diagnoses, chronic illness diagnoses), and pandemic-related data, and completed a set of validated self-report questionnaires. Participants also answered a set of questions regarding the SARS-COV-2 infection, such as associated symptoms, need for hospitalization, perception of symptom severity (rated on a scale from 0 [mild symptoms] to 10 [severe symptoms]), and concern about having infected someone (rated on a scale from 0 [“Never”] to 5 [“All the time”]) (see Table 1). The self-report questionnaires used were as follows.

#### Anxiety and depressive symptoms

Hospital Anxiety and Depression Scale (HADS) (Zigmond and Snaith, 1983). The HADS is a 14-item self-report scale with two 7-item subscales measuring anxiety (e.g., “I get sudden feelings of panic”) and depressive symptoms (e.g., “I still enjoy the things I used to enjoy”). Items are rated on a 4-point response scale (from 0 to 3) with total scores ranging from 0 to 21 for each subscale. Higher scores denote higher levels of anxiety/depression. In this study, Cronbach’s alphas of 0.85 and 0.84 were found for the anxiety and depression subscales, respectively.

#### Post-traumatic stress

Posttraumatic Symptom Disorder Checklist Civilian Version (PCL-C; Weathers et al., 1994). The PCL-C measures symptoms of PTSD, as defined by the DSM-VI-TR. This scale comprises 17 items (e.g., “Suddenly acting or feeling as if a stressful experience were happening again [as if you were reliving it]”) rated on a 5-point Likert-type scale (1: “Not at all,” 5: “Extremely”). In this study, the scale was administered by asking participants to consider their experiences relating to the SARS-CoV-2 infection. The scale presented a Cronbach’s alpha of 0.93 in our sample. Total scores of the PCL-C can range from 17 to 85 with higher scores indicating more severe PTSD symptomatology. The guidelines of the U.S. National Center for PTSD (2012) recommend the use of a cut-off of 44 to screen for PTSD in medical populations. In the current study, the Cronbach’s alpha of PCL-C was 0.93.

#### Recent major life events

Major life events questionnaire (MLEQ; Fonseca et al., 2020). The MLEQ is a checklist of 22 items (e.g., “Did someone you were close to die during the last 12 months?”), each representing a major life event (e.g., marriage, pregnancy, serious illness, financial problems). Participants are asked to report whether each event has occurred during the previous 12 months. This questionnaire has been used in previous studies studying major life events. Because it is a checklist of events, a Cronbach’s alpha has not been calculated.

#### Shame related to SARS-CoV-2 infection

Chronic Illness-Related Shame Scale (CISS), SARS-CoV-2 version (adapted from Trindade et al., 2017). The CISS is a 7-item (e.g., “I’m ashamed of talking with others about my illness or symptoms”) validated measure of chronic illness shame, rated on a 5-point Likert scale (0: “never true,” 4: “always true”). For this study, each item of the CISS was adapted to portray shame related to a SARS-CoV-2 infection. This scale presented a Cronbach’s alpha of 0.87 in the present study.

#### Resilience

Resilience Scale for Adults (RSA; Friborg et al., 2003). The RSA is a 33-item (e.g., “I know that I can solve my personal problems”) instrument that measures protective resilience factors in adults.Items are rated from 1 to 7 and higher scores reveal higher levels of protective resilience factors. In the current study, the RSA’s internal consistency was excellent (α = 0.91).

#### Psychological flexibility

Portuguese Comprehensive Assessment of Acceptance and Commitment Therapy Processes (CompACT; Francis et al., 2016; Trindade et al., 2021). The Portuguese CompACT is an 18-item (e.g., “I go out of my way to avoid situations that might bring difficult thoughts, feelings, or sensations”) self-report measure of psychological flexibility, as defined by ACT. It comprises three different dimensions: openness to experience, behavioural awareness and valued action. Items are answered on a 7-point Likert scale (1: “strongly disagree,” 6: “strongly agree”) and higher scores indicate greater psychological flexibility. In this study, CompACT’s internal consistency was acceptable (*α* = 0.78).

### Data analysis

All statistical analyses were conducted using SPSS (v. 28 SPSS, Chicago, IL, USA). Descriptive analyses were conducted to examine sociodemographic and clinical data, as well as the mean and standard deviation scores of all study variables. Chi-square (categorical variables) and independent *t*-test (continuous variables) were performed to examine group differences (completers vs. non-completers) regarding sociodemographic, clinical, pandemic-related characteristics, and psychological variables (see Supplementary Table S1). The prevalence of a possible PTSD diagnosis among the SARS-CoV-2 survivors group was assessed using the recommended PCL-C cut-off scores (PTSD NCf, 2012). Likewise, clinically meaningful depression and anxiety scores among groups were established using the cut-off score from HADS.

Person product–moment correlations (for continuous variables) and point biserial correlations (for dichotomous variables) were performed to explore cross-sectional (T1) and longitudinal (T1 and T2) associations between variables. Following the correlation analyses, longitudinal regression models were conducted to examine the predictors of PTSD, anxiety and depressive symptoms (cross-sectional regression analyses were also conducted; see Supplementary Tables S2–S4). For the longitudinal analysis, three separate stepwise linear regression models were performed with predictors (measured at T1) and PTSD, anxiety, and depressive symptoms 12 months later (measured at T2). Predictors were entered by steps considering: (1) sociodemographic and clinical variables; (2) contextual and psychological variables; and (3) psychological flexibility. We have not included symptomatology at T1 as predictors in our models given that correlation analyses suggest possible multicollinearity between symptoms measured at T1 and T2 (*r* > 0.70). Also, given that complex network analyses of the longitudinal relationship between psychopathological symptoms at one time-point on same (and other) symptoms later on (e.g., see Eaton et al., 2023 for a review), we have anticipated that results on contextual factors and psychological processes could thus be biased (i.e., falsely show non-significant effects when significant ones could exist).

## Results

### Preliminary analysis

A preliminary analysis was conducted to assess the statistical assumptions. Outliers were not found for the dependent variables (i.e., depression, anxiety, and PTSD symptoms at T2). Additionally, no severe violations of the normal distribution were found (skewness ranged from 0.50 for depression and 1.44 for PTSD symptoms; kurtosis ranged from −0.96 for depression and 1.59 for PTSD symptoms). Variance Inflation Factor (VIF) values confirmed the absence of multicollinearity for all independent variables (VIF values ranged from 1.113 for having children and 1.863 for resilience). No missing data was found given that participants had to provide complete responses to successfully submit the online form.

### Prevalence of clinically meaningful symptoms of PTSD, anxiety, and depressive symptoms

Results regarding the proportion of participants from both groups per anxiety and depressive symptoms level, and the proportion of SARS-CoV-2 survivors with a possible PTSD diagnosis at T1, can be found in Table 1. Considering the recommended PCL-C cut-off score for individuals receiving specialised medical care (PTSD NCf, 2012), 32.90% presented a possible PTSD diagnosis related to the experience of having been infected with the SARS-CoV-2 virus. Only 42.6% of the participants presented normal levels of anxiety. Concerning depression, 67.9% of the participants presented a normal level of depressive symptomatology.

### Associations with PTSD, anxiety, and depressive symptoms

Having a psychiatric diagnosis at T1 was correlated with higher levels of anxiety and depressive symptomatology 1 year later (T2). Having children at T1 was associated with more PTSD symptoms at T2, and a higher number of recent major life events and higher levels of shame related to the SARS-CoV-2 infection at T1 were positively associated with PTSD, anxiety, and depressive symptoms at T2. Resilience and psychological flexibility at T1 were negatively correlated with symptoms of anxiety and depression at T2, and psychological flexibility at T1 was correlated with PTSD at T2 (see Table 2). Table 2 also includes cross-sectional correlation results (using baseline data).

**Table 2:** Pearson correlations between sociodemographic, COVID-related characteristics, and psychological variables, and PTSD, Anxiety and Depressive Symptoms at T1 (*n* = 209) and T2 (*n* = 61).

	M ± SD	PTSD symptoms T1	Anxiety T1	Depressive symptoms T1	PTSD symptoms T2	Anxiety T2	Depressive symptoms T2
Variables at T1							
Age	35.35 ± 10.09	0.04	−0.01	−0.07	0.19	0.10	0.01
Gender (men: 0; women: 1)	–	0.15*	0.19**	0.15**	0.01	0.11	0.09
Marital status (without partner: 0; with partner: 1)	–	0.05	−0.03	−0.07	0.08	0.07	−0.08
Having children†	–	0.16*	−0.06	0.02	0.30*	0.20	0.11
Comorbid physical illness†	–	0.11	0.13	0.01	0.00	−0.03	0.04
Previous psychiatric diagnosis†	–	0.29***	0.41***	0.25***	0.22	0.40**	0.34**
Perception of COVID-19 symptom severity	4.89 ± 3.20	0.19**	0.10	0.11	0.10	0.07	−0.01
Need for hospitalization†	–	0.10	0.06	−0.04	††	††	††
Concern about having infected someone	3.26 ± 1.59	0.21**	0.14*	0.07	0.01	0.03	0.09
Duration of isolation period (in days)	18.43 ± 44.92	0.05	0.01	−0.04	−0.17	−0.09	−0.05
In isolation during assessment†	–	0.11	0.07	0.06	−0.01	−0.02	−0.05
Working from home	–	−0.15*	−0.11	−0.10	0.03	0.09	0.06
Lost job due to the pandemic†	–	0.11	0.12	0.17*	0.10	0.02	0.09
Lost someone due to COVID-19†	–	0.06	0.11	0.11	0.16	0.13	0.05
Number of recent major life events	3.76 ± 2.99	0.27***	0.38***	0.36***	0.45***	0.45***	0.53***
SARS-CoV-2 infection shame	6.42 ± 6.36	0.52***	0.45***	0.35***	0.30*	0.30*	0.43***
Resilience	171.42 ± 27.95	−0.37***	−0.48***	−0.60***	−0.22	−0.40***	−0.52***
Psychological flexibility	62.35 ± 13.27	−0.47***	−0.52***	−0.50***	−0.48***	−0.48***	−0.47***
PTSD Symptoms T1	32.9 ± 13.22	–	0.68^***^	0.59^***^	0.75^***^	0.63^***^	0.67^***^
Anxiety T1	8.83 ± 4.46	–	–	0.69^***^	0.62^***^	0.74^***^	0.71^***^
Depressive symptoms T1	6.28 ± 4.29	–	–	–	0.56^***^	0.64^***^	0.73^***^

† (yes: 1; no: 0); †† no participant from T2 required hospitalization; M, mean; SD, standard deviation. *p < 0.05; **p < 0.01; ***p < 0.001.

Significant associations of high magnitude were found between each symptomatology at T1 and at T2 (*r* > 0.70), as well as between all symptoms at T1 and at T2 (*r* > 0.60).

### Factors longitudinally explaining PTSD, anxiety, and depressive symptoms

Baseline predictors of PTSD symptoms, anxiety and depressive symptoms at follow-up were tested (see Table 3).

**Table 3:** Summary of hierarchical regression analysis for variables predicting PTSD symptoms at T2 in SARS-CoV-2 survivors (*n* = 61).

***PTSD Symptoms***	Model 1			Model 2			Model 3		
*Variables at T1*	*B*	*SE*	β	*B*	*SE*	β	*B*	*SE*	β
Having children	8.46	3.49	0.30*	8.59	3.15	0.31**	8.94	2.92	0.32**
Number of recent major life events				2.49	0.68	0.43***	1.98	0.65	0.34**
SARS-CoV-2 infection shame				0.23	0.25	0.11	0.02	0.24	0.01
Psychological flexibility							−0.33	0.10	−0.36**
*R^2^*	0.09			0.32			0.42		
*F*	5.86*			8.78***			10.22***		

Anxiety Symptoms	Model 1			Model 2			Model 3		
Variables at T1	*B*	SE	*β*	*B*	SE	*β*	*B*	SE	*β*
Previous psychiatric diagnosis	2.03	0.58	0.42***	0.81	0.64	0.17	0.68	0.62	0.14
Number of recent major life events				0.59	0.24	0.32*	0.55	0.23	0.29*
SARS-CoV-2 infection shame				0.14	0.08	0.21	0.12	0.08	0.18
Resilience				−0.01	0.02	−0.09	0.00	0.02	0.02
Psychological flexibility							−0.09	0.04	−0.29*
*R^2^*	0.17			0.35			0.40		
*F*	12.28***			7.40***			7.43***		

Depression symptoms	Model 1			Model 2			Model 3		
Variables at T1	*B*	SE	β	*B*	SE	β	*B*	SE	β
Previous psychiatric diagnosis	1.58	0.54	0.36**	0.06	0.55	0.01	−0.03	0.55	−0.01
Number of recent major life events				0.58	0.21	0.34**	0.55	0.20	0.32**
SARS-CoV-2 infection shame				0.12	0.07	0.19	0.10	0.07	0.17
Resilience				−0.04	0.02	−0.26	−0.03	0.02	−0.18
Psychological flexibility							−0.06	0.03	−0.21
*R^2^*		0.13			0.40			0.43	
*F*		8.64**			9.41***			8.40***	

*p < 0.05, **p < 0.01, ***p < 0.001.

To longitudinally examine the predictors (measured at T1) of PTSD symptoms severity measured 1 year later (T2) (see Table 3), considering the significant correlations, a regression model was tested. Having children was the only independent variable included in the first step (Model 1) of the regression analysis, which resulted in a significant effect. In the second step of the analysis, the number of recent major life events and shame related to the SARS-CoV-2 infection were added to the model (Model 2). Having children and number of major life events were significant predictors, and accounted for 32% of the variance. Psychological flexibility was added to the final model, and accounted for further 10% of the variance of PTSD symptoms severity. In the final model (Model 3), this outcome was predicted by having children, a high number of recent major life events, and low psychological flexibility, measured 1 year prior, which explained 42% of the total of variance of PTSD symptoms.

To longitudinally analyse the predictors of anxiety at T2, the variable “having a previous psychiatric diagnosis” was added in the first step of the regression model (Model 1), with a significant effect. In the second step, the number of recent major life events, SARS-CoV-2 infection-related shame, and resilience were added (Model 2). Only a high number of prior major life events was a significant predictor of the model at this stage (*R*^2^ = 0.35). In the final step of the regression, after adding psychological flexibility, a high number of major life events and low psychological flexibility predicted anxiety 1 year later, in a model that explained 40% of this outcome (Model 3).

Regarding depressive symptoms, results showed that having a previous psychiatric diagnosis at T1 was a significant predictor of depressive symptoms at T2, in the first step of the analysis (Model 1). Only a higher number of major life events was a significant predictor of depressive symptoms at both the second and third models of the regression model, even after the addition of psychological flexibility, which was a non-significant predictor (*p* = 0.088). The final model (Model 3) explained 43% of the variance in depressive symptoms at T2.

## Discussion

This study examined the long-term impact of psychosocial factors on symptoms of PTSD, anxiety, and depression in a sample of Portuguese SARS-CoV-2 survivors. Results showed that SARS-CoV-2 survivors present high symptoms of depression and anxiety: approximately 60% present mild to severe symptoms of anxiety, and approximately 35% present symptoms of depression. Pre-pandemic normative data in community samples (Breeman et al., 2015) suggest that more than 80% present normal levels of anxiety and depression (see Zigmond and Snaith, 1983 for cut-off scores). The elevated symptoms of anxiety and depression seem to echo previous results on the nefarious impact of the COVID-19 pandemic on the population’s mental health (Penninx et al., 2022). Results of this study also showed that 42% of SARS-CoV-2 survivors present clinically significant symptoms that support a possible diagnosis of PTSD. This result is in accordance with many previous studies that found PTSD symptoms during the COVID-19 pandemic (Giannopoulou et al., 2021), including in multicultural samples (Matos et al., 2021a), although higher than found in other studies (Yuan et al., 2021). In addition to contextual stressors related to the pandemic and/or its mitigation measures (e.g., our sample was more than 2 weeks in isolation, 8.1% lost jobs during the pandemic, and 10.5% lost someone due to COVID-19), intrapersonal psychological processes may have contributed to the elevated symptoms of PTSD, anxiety, and depression. This seems to be the case in our sample, in which, although the majority of participants have recovered from the infection without prescribed medication (62.2%) (which suggests mild illness), they nonetheless showed regular concern for infecting others (65.9%).

Several regression models were tested according to the significance of the longitudinal correlations to test the contextual and psychological factors longitudinally predicting PTSD, anxiety, and depression. Having children, as well as having more recent major life events, were significant predictors of PTSD symptoms measured 1 year later. This goes in line with what cross-sectional literature has shown regarding the parental psychopathological symptoms during the COVID-19 pandemic, where parents reported struggles in balancing professional demands, homeschooling children, and overall domestic tasks (Davis et al., 2021). This seems to suggest that the traumatic effects of the pandemic are not exclusively experienced by parents of most vulnerable children (Guideline NG155 N, 2020) but rather by parents in general, who seem to experience long-term PTSD symptoms more than those without children. Although studies on the impact of major life events on mental health during the pandemic have been scarcely explored, our results seem to resonate with cross-sectional literature on the effects of daily hassles (e.g., unemployment, family discord) and stressful life events (e.g., economic, job, and housing difficulties) on mental health (Rossi et al., 2021).

In addition to these contextual factors, psychological flexibility was a significant predictor of PTSD symptoms. Consistent with previous studies (McCracken et al., 2022), this suggests that the ability to flexibly choose to maintain or change behavior according to environmental cues and personal values, particularly during the second lockdown (when our baseline assessment was conducted), was a protective factor against PTSD symptoms. This was also the case for anxiety symptoms: not only those who experienced fewer major life events during the pandemic, but also those who were more psychologically flexible, presented lower levels of anxiety 1 year later. These results seem to add to those findings by providing a longitudinal picture of the protective role of psychological flexibility against psychopathological symptoms during the pandemic. Interestingly enough, when it comes to depressive symptoms, major life events were the only significant predictor. While PTSD and anxiety, by their evolutionary programming (see Price, 2003), might involve overstimulation of threat/protection-focused systems (including psychological processes that activate “better safe than sorry” behavioral rules inherent in psychological inflexibility), depression is hypothesized to lay on other evolutionary routes, such as defeat and entrapment (Gilbert and Allan, 1998), which major life events such as unemployment, divorce, and loss may activate. In fact, major stressful life events are well-known predictors of depression (Tennant, 2002).

It should be noted that neither covid-related shame nor resilience had a significant long-term impact on symptoms of PTSD, anxiety, and depression. These results seem to indicate that although lower levels of shame and higher levels of resilience were associated with better mental health indicators during lockdown (T1), their long-term impact was not significant when other contextual and psychological variables were controlled. It may be the case that resilience is a more important factor in other symptomatology, perhaps more salient than PTSD, such as adjustment-related symptoms and disorder (for a discussion, see (Brunet et al., 2022)).

## Limitations of the present study

This study was not short of limitations, which should be considered when interpreting these results. Firstly, our sample was not balanced in terms of gender, given that the majority of participants were women. A different pattern of associations and predictions might be present when considering other genders. Also, the majority of our sample was entirely cisgender and binary, which precludes us from extrapolating to queer and gender non-conforming individuals whose mental health and financial resources seem to have been significantly impacted by the pandemic (Nowaskie and Roesler, 2022). We have also not accounted for specific socioeconomic variables, such as income, which might be a buffer of the family burden resulting from the pandemic mitigation measures. Additionally, sample selection bias due to online data collection should be acknowledged. Although studies suggest that self-report measures are psychometrically equivalent in online and paper-and-pencil data collections (Trindade et al., 2021), the generalizability of results is unwarranted, given that online data collection might yield bias due to unattentiveness and lack of sociodemographic representativeness. Also, the high attrition rate (70.83%) should be considered when interpreting results, as these might reflect a biased self-selected sample. Additionally, the fact that we have collected data in two-time points instead of multiple ones (>3) prevents us from conducting much-needed trajectory analyses that would provide a picture on changes of each variable over time. Future studies, with larger sample sizes that would ensure sufficient statistical power to test structural equation modeling, should conduct latent growth curve model analyses to explore the rate of changes over time (the slope factor). Also noteworthy is the fact that we have yet to explore indirect or interaction effects underlying the relationship between psychopathological symptoms at different time points. Future studies with sufficiently large sample sizes that would not compromise statistical power should explore these relationships, not only through mediational and moderation analyses, but also through network analyses that would provide evidence on the complex interconnection and inter-dependence between symptoms, contextual factors, and psychological processes. Finally, these results should consider the specificities of governmental measures before a cross-national generalization. The Portuguese government implemented strict measures to mitigate the pandemic compared to other European countries, which might have had a specific considerable impact on financial loss and mental health.

## Conclusion

Overall, these results seem to suggest the importance of considering both contextual and psychological variables when developing comprehensive models, as well as mental health prevention and intervention programs, during periods of global pandemic. These results suggest the long-term detrimental effects of pandemic periods on PTSD, anxiety, and depression, as well as point out the predictive role of parenting, major life events, and psychological flexibility. This seems to suggest that measures promoting mental health during pandemic crises should focus on reducing parental burden and socioeconomic losses, as well as implementing evidence-based programs aiming to promote protective psychological skills, such as psychological flexibility (e.g., Acceptance and Commitment Therapy).

## Data Availability

The raw data supporting the conclusions of this article will be made available by the authors, without undue reservation.
