# Supplementary material for: One-year predictors of PTSD symptoms, anxiety, and depression in SARS-CoV-2 survivors: psychological flexibility and major life events as main predictive factors
Source: Front Psychol. 2024 Aug 27;15:1378213. doi: 10.3389/fpsyg.2024.1378213 (PMC11385856; doi:10.3389/fpsyg.2024.1378213)
Supplement: Supplementary file 1 [file Table_1.docx]

| **Supplementary Table 1**  *Comparisons Between SARS-CoV-2 Survivors (N = 209) Completers and Non-completers at T1* | | | | |  |
| --- | --- | --- | --- | --- | --- |
|  | Completers  (*n* = 61) | Non-completers  (*n* = 148) | Test of differences | *p* | |
| Gender, % |  |  | 1.18 | .279 | |
| Men | 9.8 | 15.5 |  |  |  |
| Women | 90.2 | 84.5 |  |  |  |
| Age, M ± SD | 36.62 (11.88) | 34.82 (9.25) | -1.06 | .293 | |
| Marital Status, % |  |  | 7.88 | .049 | |
| Single | 57.4 | 40.5 |  |  |  |
| Married or cohabiting | 37.7 | 55.4 |  |  |  |
| Divorced | 3.3 | 4.1 |  |  |  |
| Widowed | 1.6 | 0 |  |  |  |
| Have children, % † | 44.3 | 49.3 | 0.44 | .505 | |
| Employment status, % |  |  | 4.06 | .398 | |
| Employed | 70.5 | 79.7 |  |  |  |
| Unemployed | 14.8 | 10.1 |  |  |  |
| Student | 11.5 | 8.8 |  |  |  |
| Working student | 1.6 | 1.4 |  |  |  |
| Retired | 1.6 | 0 |  |  |  |
| Working from home, % † | 49.2 | 29.1 | 7.70 | .006 | |
| Previous psychiatric diagnosis, % † | 31.1 | 30.4 | 0.01 | .916 | |
| Comorbid physical illness, % † | 14.8 | 23 | 1.79 | .181 | |
| Days in isolation, M ± SD | 12.80 (18.43) | 15.11 (28.34) | 0.59 | .557 | |
| Lost job due to pandemic, % † | 8.2 | 8.1 | 0.00 | .983 | |
| Lost someone to COVID-19, % † | 11.5 | 10.1 | 0.08 | .774 | |
| Vaccinated, % † | 1.6 | 3.4 | 0.47 | .494 | |
| Perception of COVID symptoms severity, M ± SD | 4.39 (3.18) | 5.10 (3.20) | 1.46 | .147 | |
| Total of symptoms, M ± SD | 4.07 (2.76) | 3.88 (2.56) | -0.47 | .639 | |
| Need for medication, % † | 16.4 | 14.9 | 0.08 | .780 | |
| Need for hospitalization, % † | 1.6 | 4.1 | 0.78 | .378 | |
| Concern about infecting someone, M ± SD | 3.11 (1.57) | 3.32 (1.60) | 0.84 | .402 | |
| Being in isolation during assessment, % † | 13.1 | 18.9 | 1.02 | .312 | |
| No. of recent major life events, M ± SD | 3.59 (2.43) | 3.82 (3.19) | 0.51 | .607 | |
| Psychological flexibility, M ± SD | 64.79 (15.24) | 61.35 (12.29) | -1.56 | .121 | |
| PTSD symptoms, M ± SD | 31.30 (12.88) | 33.56 (13.34) | 1.13 | .261 | |
| Anxiety symptoms, M ± SD | 8.85 (4.43) | 8.61 (4.27) | -0.37 | .711 | |
| Depressive symptoms, M ± SD | 5.72 (4.08) | 5.84 (4.09) | 0.19 | .852 | |
| Resilience, M ± SD | 175.11 (28.34) | 169.89 (27.73) | -1.23 | .220 | |
| SARS-CoV-2 infection shame, M ± SD | 1.38 (0.53) | 1.33 (0.48) | -0.70 | .484 | |
| *Note*. † (yes: 1; no: 0). M = Mean; SD = Standard deviation. Differences were tested using independent samples t-tests (continuous variables) and chi-square test (categorical variables). | | | | | |
