# Supplementary material for: One-year predictors of PTSD symptoms, anxiety, and depression in SARS-CoV-2 survivors: psychological flexibility and major life events as main predictive factors
Source: Front Psychol. 2024 Aug 27;15:1378213. doi: 10.3389/fpsyg.2024.1378213 (PMC11385856; doi:10.3389/fpsyg.2024.1378213)
Supplement: Supplementary file 2 [file Table_2.docx]

**Supplementary Table 2**

*Summary of Hierarchical Regression Analysis (Cross-sectional) for Variables Predicting PTSD, Anxiety and Depression Symptoms at T1 (N = 209)*

| **PTSD Symptoms** | Model 1 | | | Model 2 | | | Model 3 | | |
| --- | --- | --- | --- | --- | --- | --- | --- | --- | --- |
| *Variables at T1* | *B* | *SE* | β | *B* | *SE* | β | *B* | *SE* | β |
| Gender | 4.38 | 2.41 | .12 | 3.84 | 2.08 | .10 | 4.55 | 1.99 | .12* |
| Working from home | -3.29 | 2.41 | -.12 | -2.14 | 1.55 | -.08 | -1.26 | 1.49 | -.05 |
| Having children | 2.70 | 1.73 | .10 | 3.96 | 1.54 | .15* | 3.31 | 1.48 | .13* |
| Previous psychiatric diagnosis | 8.07 | 1.82 | .28*** | 2.83 | 1.70 | .10 | 2.20 | 1.62 | .08 |
| Perception of COVID-19 symptom severity | 0.52 | 0.27 | .13 | 0.42 | 0.24 | .10 | 0.41 | 0.23 | .10 |
| Concern about having infected someone | 1.75 | 0.53 | .21** | 1.02 | 0.46 | .12* | 0.68 | 0.45 | .08 |
| Number of recent major life events |  |  |  | 0.63 | 0.26 | .14* | 0.64 | 0.25 | .15* |
| SARS-CoV-2 infection shame |  |  |  | 0.73 | 0.13 | .35*** | 0.71 | 0.12 | .34*** |
| Resilience |  |  |  | -0.09 | 0.03 | -.19** | -0.02 | 0.03 | -.04 |
| Psychological flexibility |  |  |  |  |  |  | -0.29 | 0.06 | -.30*** |
| *R^2^* | .20 | | | .42 | | | .47 | | |
| *F* | 8.49*** | | | 15.73*** | | | 17.68*** | | |
| \| **Anxiety Symptoms** \| Model 1 \| \| \| Model 2 \| \| \| Model 3 \| \| \| \| --- \| --- \| --- \| --- \| --- \| --- \| --- \| --- \| --- \| --- \| \| *Variables at T1* \| *B* \| *SE* \| β \| *B* \| *SE* \| β \| *B* \| *SE* \| β \| \| Gender \| 1.90 \| 0.77 \| .15* \| 1.78 \| 0.66 \| .14** \| 2.01 \| 0.61 \| .16** \| \| Previous psychiatric diagnosis \| 1.94 \| 0.29 \| .42*** \| 0.99 \| 0.27 \| .21*** \| 0.88 \| 0.25 \| .19*** \| \| Concern about having infected someone \| 0.38 \| 0.17 \| .14* \| 0.18 \| 0.15 \| .07 \| 0.05 \| 0.14 \| .02 \| \| Number of recent major life events \|  \|  \|  \| 0.31 \| 0.08 \| .21*** \| 0.31 \| 0.07 \| .22*** \| \| SARS-CoV-2 infection shame \|  \|  \|  \| 0.16 \| 0.04 \| .23*** \| 0.15 \| 0.04 \| .22*** \| \| Resilience \|  \|  \|  \| -0.04 \| 0.01 \| -.28*** \| -0.02 \| 0.01 \| -.11 \| \| Psychological flexibility \|  \|  \|  \|  \|  \|  \| -0.11 \| 0.02 \| -.36*** \| \| *R^2^* \| .22 \| \| \| .44 \| \| \| .52 \| \| \| \| *F* \| 19.59*** \| \| \| 26.89*** \| \| \| 30.95*** \| \| \| \| \| **Depression Symptoms** \| Model 1 \| \| \| Model 2 \| \| \| Model 3 \| \| \| \| --- \| --- \| --- \| --- \| --- \| --- \| --- \| --- \| --- \| --- \| \| *Variables at T1* \| *B* \| *SE* \| β \| *B* \| *SE* \| β \| *B* \| *SE* \| β \| \| Gender \| 1.55 \| 0.78 \| .13* \| 1.45 \| 0.62 \| .12* \| 1.58 \| 0.60 \| .13** \| \| Previous psychiatric diagnosis \| 1.11 \| 0.29 \| .25*** \| 0.11 \| 0.25 \| .03 \| 0.05 \| 0.24 \| .01 \| \| Lost job due to the pandemic \| 2.25 \| 0.98 \| .15* \| 0.45 \| 0.82 \| .03 \| 0.62 \| 0.80 \| .04 \| \| Number of recent major life events \|  \|  \|  \| 0.28 \| 0.08 \| .21*** \| 0.30 \| 0.09 \| .21*** \| \| SARS-CoV-2 infection shame \|  \|  \|  \| 0.08 \| 0.04 \| .12* \| 0.07 \| 0.04 \| .10 \| \| Resilience \|  \|  \|  \| -0.07 \| 0.01 \| -.50*** \| -0.06 \| 0.01 \| -.38*** \| \| Psychological flexibility \|  \|  \|  \|  \|  \|  \| -0.07 \| 0.02 \| -.24*** \| \| *R^2^* \| .10 \| \| \| .43 \| \| \| .47 \| \| \| \| *F* \| 8.77*** \| \| \| 27.35*** \| \| \| 27.25*** \| \| \| \| *Note*. **p* < .05, ***p* < .01, ****p* < .001 \| \| \| \| \| \| \| \| \| \| \| \| \| \| \| \| \| \| \| \| | | | | | | | | | |
